# Drug target affinity prediction based on multi-scale gated power graph and multi-head linear attention mechanism

**DOI:** 10.1371/journal.pone.0315718

**Published:** 2025-02-21

**Authors:** Shuo Hu, Jing Hu, Xiaolong Zhang, Shuting Jin, Xin Xu

**Affiliations:** 1 School of Computer Science and Technology, Wuhan University of Science and Technology, Wuhan, Hubei, China; 2 Hubei Province Key Laboratory of Intelligent Information Processing and Real-Time Industrial System, Wuhan, China; 3 Institute of Big Data Science and Engineering, Wuhan University of Science and Technology, Wuhan, Hubei, China; Nanyang Technological University, SINGAPORE

## Abstract

For the purpose of developing new drugs and repositioning existing ones, accurate drug-target affinity (DTA) prediction is essential. While graph neural networks are frequently utilized for DTA prediction, it is difficult for existing single-scale graph neural networks to access the global structure of compounds. We propose a novel DTA prediction model in this study, MAPGraphDTA, which uses an approach based on a multi-head linear attention mechanism that aggregates global features based on the attention weights and a multi-scale gated power graph that captures multi-hop connectivity relationships of graph nodes. In order to accurately extract drug target features, we provide a gated skip-connection approach in multiscale graph neural networks, which is used to fuse multiscale features to produce a rich representation of feature information. We experimented on the Davis, Kiba, Metz, and DTC datasets, and we evaluated the proposed method against other relevant models. Based on all evaluation metrics, MAPGraphDTA outperforms the other models, according to the results of the experiment. We also performed cold-start experiments on the Davis dataset, which showed that our model has good prediction ability for unseen drugs, unseen proteins, and cases where neither drugs nor proteins has been seen.

## Introduction

Finding compounds that can attach to specific targets in a selective manner and produce desired effects is the main goal of drug discovery. Central to this pursuit is the prediction of DTA, a critical stage that guides subsequent research efforts. Drug discovery using traditional methods is an expensive and lengthy procedure, usually lasting 10–15 years from screening to approval and costing billions of dollars [1]. In contrast, drug repositioning has received more and more attention and made great progress due to its features of fast speed and low cost [2]. Precisely identifying interactions between drugs and targets holds paramount importance in drug repositioning efforts, where affinity serves as a pivotal measure for evaluating the strength of these interactions. Owing to the quick advancement of computer technology, in silico methods for DTA prediction are becoming more and more important because they can realize DTA prediction efficiently and cost-effectively [3]. Current computational approaches for forecasting drug-target affinity (DTA) fall into two primary classes: conventional machine learning methodologies and advanced deep learning approaches.

The machine learning based approach is used for DTA prediction by combining classical models of machine learning, such as the method KronRLS [4] proposed by Pahikkala et al. which is based on kronecker regularization and least squares algorithm. In this method Smith-Waterman algorithm and PubChem structural clustering tool were used for constructing similarity matrix between drugs and proteins and then predicting DTA by kronecker product of similarity matrix. Gradient boosting is used by He et al. to extract features from drugs and targets in their proposed Simboost [5]. However, machine learning-based methods often rely on complex feature engineering in order to achieve good performance, different feature representations may lead to different performance results, and a large amount of high-level domain expertise is often required in order to find the optimal feature representations [6].

In recent years, deep learning methods [7–9] have been extensively used in the area of drug-target affinity prediction [10–12]. For example, two CNN blocks are used by DeepDTA [13] to extract feature information from protein sequences and drug SMILES, respectively. These obtained feature representations are then connected and fed into multiple fully connected layers for predicting DTA. WideDTA [14] considers four textual messages related to drugs and proteins and uses four CNN blocks for feature extraction, resulting in better performance than the previously proposed DeepDTA.

When it comes to DTA prediction, CNN-based models have demonstrated exceptional performance, however, these models only represent drugs as strings, which limits our ability to obtain structural information about the drug and may affect the model’s prediction accuracy. Graph neural networks (GNN) have been utilized for predicting DTA in order to solve this problem and significant performance improvements have been obtained. As an illustration, Nguyen et al. introduced GraphDTA [15], which uses the RDKit tool to encode drug SMILES into graphs, and then evaluates the model’s performance on four GNNs, including GIN, GAT, GCN, and GAT-GCN. According to the results of experiments, the model’s prediction ability can be further enhanced by presenting drugs as graphs. Yang et al. developed MGraphDTA, a model that constructs a drug molecule feature extraction module consisting of 27 layers of GNNs for extracting multi-scale structural features of drug molecules. DGraphDTA [16] was proposed by Jiang et al., which predicts contact maps from amino acid sequences and constructs protein graphs from them, so that both drugs and proteins are represented as graphs, and then two GNN blocks are used for feature extraction, and the model performance is further improved. WGNN-DTA [17] is a variant of DGraphDTA, and WGNN-DTA removes difficult procedures like multiple sequence alignment (MSA) in generating protein contact maps, which effectively improves the speed of modeling.

Most current graph-based DTA prediction models use 3 to 4 layers of GNNs, however, shallow GNNs do not capture the global topology of the molecular graph, resulting in limited model performance. The global structure of the graph may be further captured by using several stacked GNNs, but this leads to problems such as gradient vanishing and feature degradation. Commonly, DTA prediction models only consider direct connections between neighboring nodes and ignore indirect relationships with other nodes, which may not be effective in obtaining complex global features of the graph.

To tackle the aforementioned difficulties, we introduce MAPGraphDTA, an innovative model for DTA prediction. The model uses a multi-head linear attention (MHLA) mechanism and a multi-scale power graph for feature extraction. We use three GCN blocks with inputs M, M^2^, and M^3^ (PMGCN) and a multi-scale CNN block with a MHLA mechanism (AMCNN) to get the feature representations of drugs and targets, respectively. Through the introduction of power graphs that consider the multi-hop connectivity connection of molecular graphs, we are able to acquire an abundance of global features in the drug feature extraction module PMGCN. To leverage this information more effectively, we integrate a gated skip-connection mechanism into the GNN, which can fuse features of different scales and can effectively deal with problems such as gradient vanishing and feature degradation. We use multi-scale CNN blocks in the protein feature extraction module AMCNN to obtain comprehensive and rich protein feature representations, and in order that important features are not neglected, we provide a new MHLA mechanism that selectively concentrates on the whole biological sequence and then selectively aggregates global feature representations according to the calculated attention weights, which is conducive to the further improvement of the performance of our model. Our model performs better than other cutting-edge models across all evaluation metrics, according to our experiments on several of datasets.

## Methods

### Model architecture

**Fig 1:**
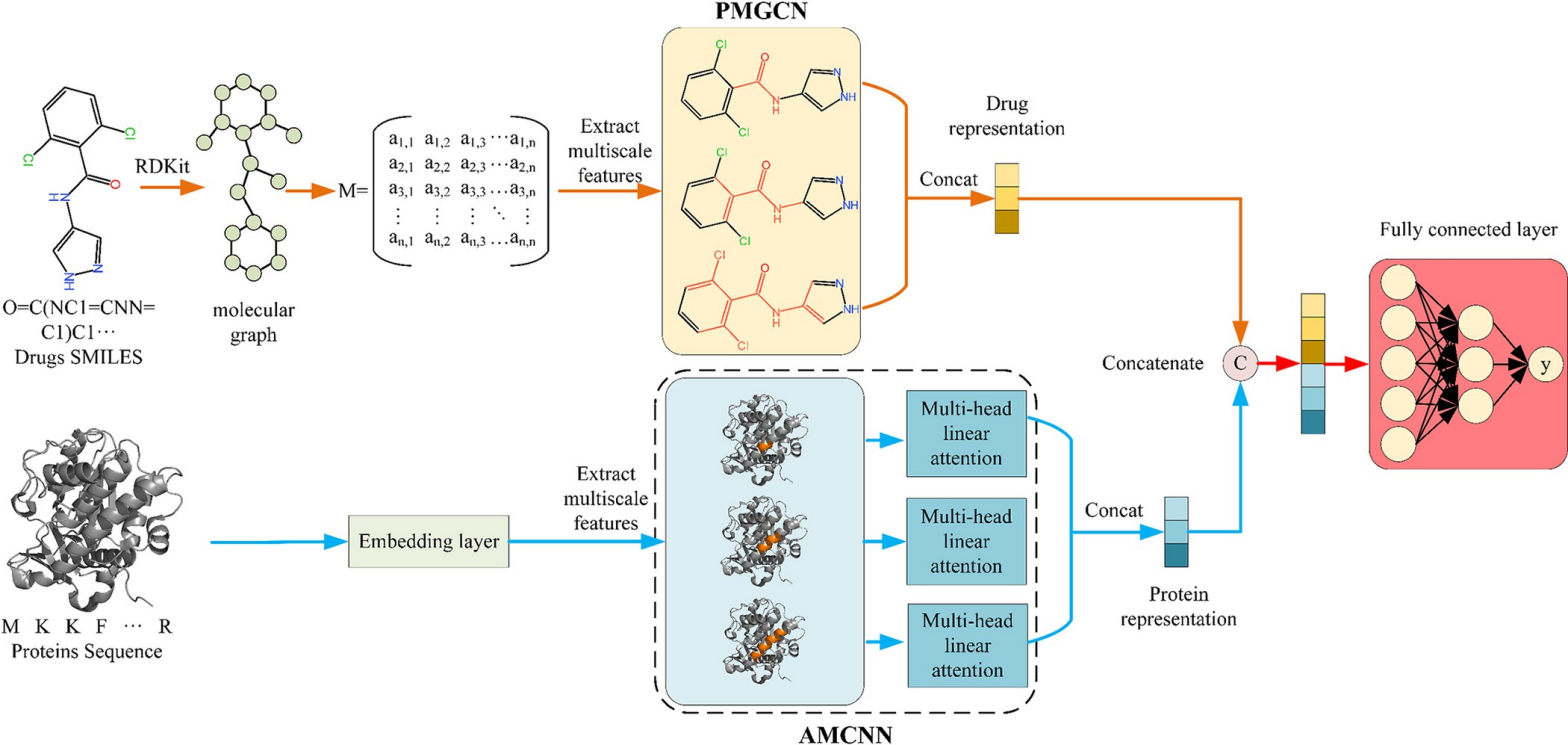
Overview architecture of the MAPGraphDTA model.

The MAPGraphDTA model’s basic design is shown in Fig 1. The model has two main functional modules, in the first module PMGCN, we use the drug SMILES as the original input and construct the drug molecule graph, then we obtain the multi-hop connectivity relationship of the drug molecules through the power graph, and finally we use a multi-scale GCN to extract the drug features. The second module, AMCNN, utilizes the amino acid sequence of a protein as its primary input, then map the amino acid sequence to an integer sequence based on the integer coding of the amino acid sequence, and finally input it into a multi-scale CNN with a MHLA mechanism to extract protein features. For creating a unified representation, we combine the feature representations of both drugs and proteins. Following this, the merged representation undergoes processing through multiple fully connected (FC) layers to predict the DTA. In the following sections, we will offer an elaborate explanation of each functional module.

### Input representation

**Fig 2:**
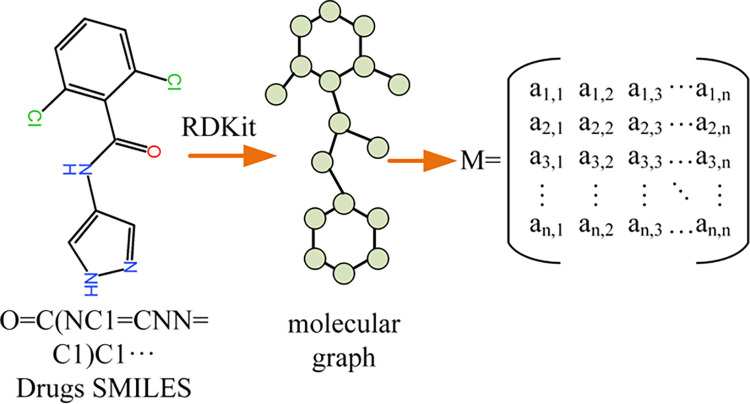
Conversion of drug SMILES to molecular graph and adjacency matrix.

We represent drug molecules using SMILES (Simplified Molecular Linear Input Specification) [18]. Meanwhile, we used protein sequences to represent target proteins, where each character represents an amino acid. It is worth noting that utilizing the SMILES string alone for representing a drug molecule lacks structural information. Therefore, we employ RDKit [19] to transform the drug SMILES into the respective drug molecule graph, as depicted in Fig 2.

For protein sequences, we map each amino acid to an integer (e.g., glutamic acid (E) is 4, alanine (A) is 1, etc.), This allows the original protein sequence to be expressed as an integer sequence. In this paper we establish the maximum length of proteins to 1000, and then map each amino acid into a 128-dimensional learnable vector through the embedding layer.

### Multiscale gated power graph for drug encoding

**Fig 3:**
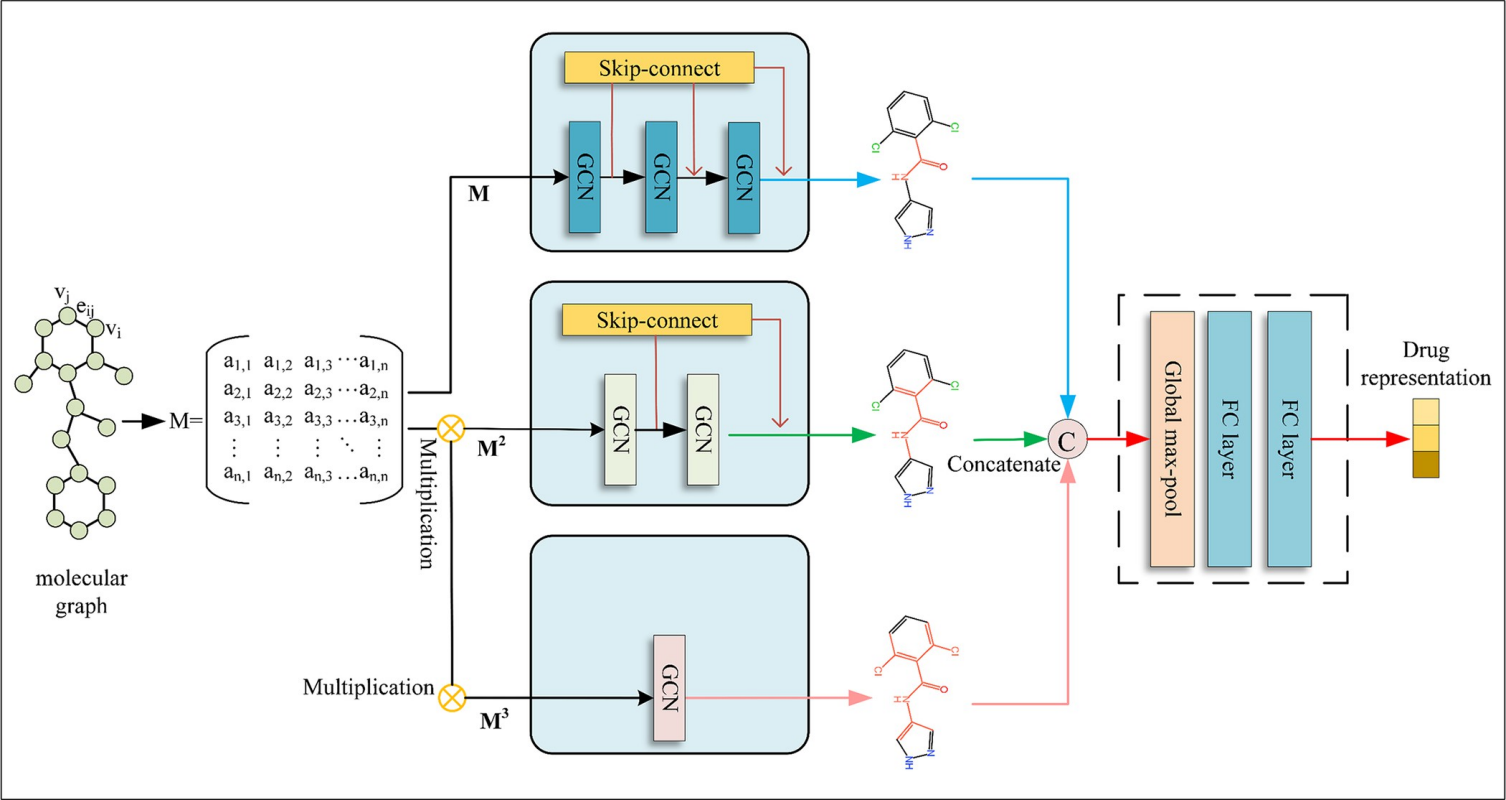
Gated Power Graph Module (PMGCN) architecture.

After representing a drug compound as a molecular graph, it is important to understand the interactions of individual nodes with their neighboring nodes in order to predict drug-target affinity scores, but considering only the connectivity between directly adjacent nodes may not fully capture the overall characteristics of the graph. In order to obtain global features of drug molecules, we designed a feature extraction module PMGCN based on power graphs and gated skip connection mechanism, which can effectively learn from graph data. Fig 3 illustrates the specific design of PMGCN, which mainly consists of three convolutional blocks with different scales, and the input of each convolutional block is a power graph with different powers, such a structure can effectively obtain the multi-hop connectivity relationship, which not only takes into account the direct action between nodes, but also can take into account the indirect correlation between them. In order to obtain drug molecular graph features more accurately, we also incorporated a gated skip-connection mechanism.

#### Power graph

Complex graph data could not fully convey the global features of the graph by considering only the relationships of neighboring nodes. By considering multi-hop connectivity relations, we can obtain indirect correlations between nodes and more distant nodes. With this approach we can obtain more abundant graph data, and thus be able to better represent the global characteristics of the graph. Motivated by the research of Mukherjee et al. [20], we use power graphs into our model to further extract the drug molecule features.

In the molecular graph, a node v is directly connected to a node in its neighborhood R(v) through an edge, and u is a node in R(v). The shortest distance between a node w in u’s neighborhood R(u) and a node v in R(v) is 2, which means that they are 2 hops apart. If every node two hops distant from v is connected to it, then the graph is called a power-of-2 graph, which we denote by M2. In this way, we can make node v connected to more distant nodes by increasing the value of the index, which enhances the local reachability of v, but at the same time makes the graph complex and dense. In general, for reasons of computational efficiency and practical applications, the shortest paths exceeding 3 hops are usually excluded when describing the structure of the drug molecular graph, so increasing the index of the power graph to more than 3 does not have a significant impact on the reachability of most nodes, and does not contribute significantly to the performance of the model.

In this paper we capture the connectivity relationship between nodes through three GCN blocks. As shown in Fig 3, we stacked 3 GCN layers in the first block to extract the features of the power graph. The second block has 2 layers of GCN stacked in it to extract the features of the power-of-2 graph. Only one layer of GCN is used in the third block to extract the features of the power-of-3 graph. In the first GCN block, we process the drug compounds using RDKit to obtain the adjacency matrix M and the feature matrix X, and then compute M and X as inputs in the first GCN block. The degree normalization issue is essential in graph neural network applications to enhance the model’s stability and performance. Eq (1) shows the normalized adjacency representation M_n_, which is computed using the following formula to solve the degree normalization issue [21] for the adjacency representation. The degree matrix of M is represented by D. In order to make the first block feasible, we use Eq (2) to compute the global representation $H_{e}^{i}$ generated at the *i*^th^ layer of the GCN with respect to M. The output of the *i*^th^ layer is $H_{e}^{0}$ = X, the trainable weight is W, and the nonlinear activation function is σ.


$$M_{n}=D^{-1/2}MD^{-1/2}$$ (1)

$$H_{e}^{i}=\sigma (M_{n}H_{e}^{\left(i-1\right)}W^{\left(i-1\right)})$$ (2)

In the second GCN block, we use the square of the adjacency matrix M (M^2^) and the feature matrix X as inputs, and similarly to the above computation, we compute the normalized adjacency representation $M_{n}^{2}$ by using Eq (3). where D´ represents M^2^’s degree matrix. Similar to the first GCN block, in order to make the second GCN block feasible, we use Eq (4) to compute the global representation $H_{e}^{\prime i}$ generated at the *i*^th^ GCN layer with respect to M^2^. σ is the nonlinear activation function, W is the trainable weights, and $H_{e}^{\prime 0}$ = X is the output of the *i*^th^ layer.


$$M_{n}^{2}=D^{\prime -1/2}M^{2}D^{\prime -1/2}$$ (3)

$$H_{e}^{\prime i}=\sigma (M_{n}^{2}H_{e}^{\prime (i-1)}W^{\prime (i-1)})$$ (4)

Similarly to the first and second blocks, we can obtain the normalized adjacency representation $M_{n}^{3}$ of M^3^ and the global representation $H_{e}^{\prime \prime i}$ about M^3^ produced by the *i*-th layer of the GCN. Then we connect the output representations of the three GCN blocks so that we get the output feature representation H_e_ of the drug compound after PMGCN processing, as shown in Eq (5). Finally, we feed H_e_ into the global max pooling layer and several FC layers, which generates the final drug representation.


$$H_{e}=concat(H_{e}^{i},H_{e}^{\prime i},H_{e}^{\prime \prime i})$$ (5)

#### Gate skip connection mechanism

In order to be able to capture drug molecule graph level features at a deep level, we stacked multiple GCN layers of different scales in the PMGCN However, in general, stacking multiple GCN layers may lead to problems such as gradient vanishing and node degradation. In order to respond this challenge effectively, we include a gated skip-connection mechanism into each hidden layer’s representation learning procedure. [22]. By varying the dropout rate and update rate, the technique may combine features from different hidden states. In our model, as the quantity of stacked GCN layers rises, through the gated skip-connection mechanism, each node not only aggregates the information carried by the remote nodes, but also retains the node’s own unique feature information. Moreover, the gated skip- connection mechanism introduces a gating mechanism where the network can perform appropriate nonlinear transformations during the information transfer process, which is beneficial for the network to learn more complex node features. Eqs (6) and (7) describe the gated skip connection mechanism.


$$Z_{i}=sigmoid(U_{1}H_{i}^{\left(l+1\right)}+U_{2}H_{i}^{\left(l\right)}+b)$$ (6)

$$H_{i}^{\left(l+1\right)}=Z_{i}H_{i}^{\left(l+1\right)}+(1-Z_{i})H_{i}^{\left(l\right)}$$ (7)

U_1_ and U_2_ denote the trainable parameters, with *b* representing a bias term. $H_{i}^{\left(l\right)}$ signifies the feature vector of node *i* in the *l*th layer, while $H_{i}^{\left(l+1\right)}$ indicates the feature vector of node *i* in the *l*+1th layer. Z_i_ represents the coefficient for the learning rate.

### Multiscale convolutional neural networks for target encoding

**Fig 4:**
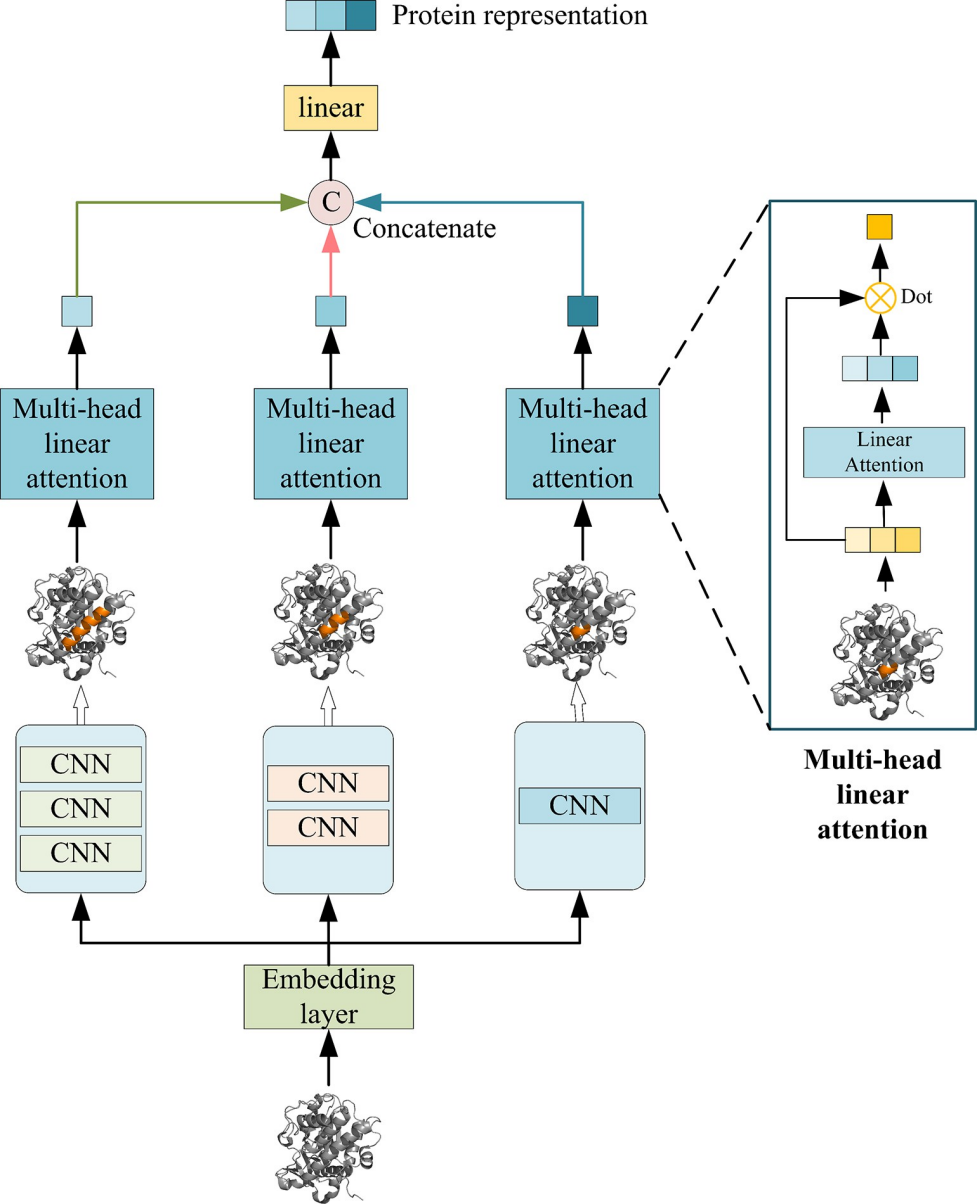
Structure of AMCNN.

To extract features from target proteins across various scales and depths, we used an AMCNN module consisting of three CNN blocks and a MHLA mechanism, as shown in Fig 4. Among them, CNN blocks of different scales have different receptive fields, and compared with the ordinary 1D CNN, our model is able to obtain more abundant information, and thus can predict the DTA more accurately. We also use a novel multi-head linear attention mechanism in this module, which enables our model to aggregate more important features and helps to improve the performance of the model.

#### Multiscale convolutional neural networks

Based on the idea of PMGCN, we used a functional module AMCNN consisting of three CNN blocks to extract protein features. As shown in Fig 4, by stacking 3*3 CNN layers, we create three CNN blocks with different receptive fields. The receptive fields of the three blocks are 3, 5, and 7, respectively. In this way, different blocks can extract protein feature information at different scales. The obtained outputs are then passed into the multi-head linear attention mechanism separately and we get the output feature representations of the three CNN blocks, and finally we connect these three output feature representations to get the final protein feature representation.

#### Multi-head linear attention mechanism

**Fig 5:**
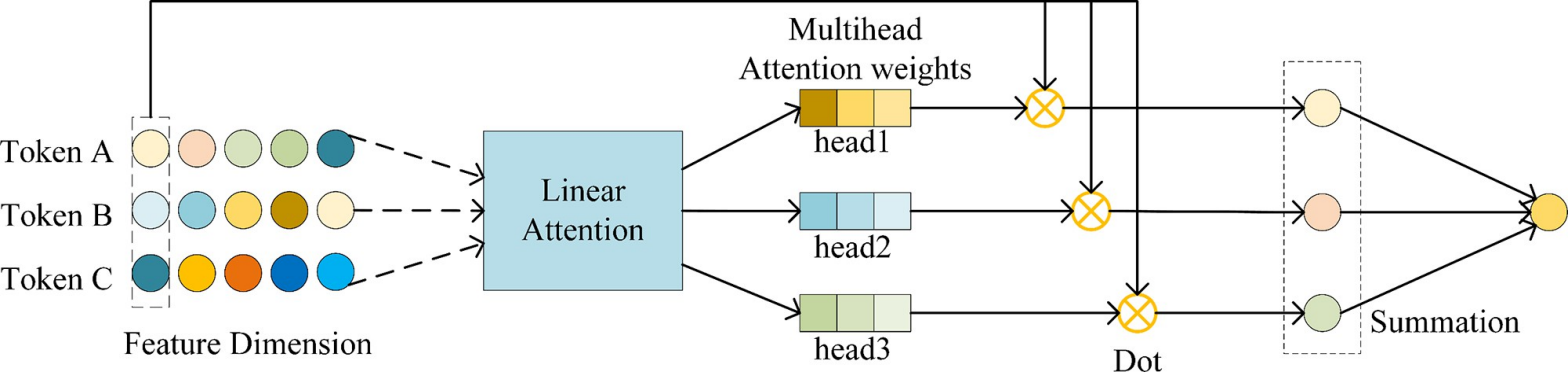
Multi-head linear attention mechanism aggregation process.

Through the multi-scale CNN, we obtain abundant feature representation, and some crucial information could be lost as a result of the traditional aggregate, so in order to be able to effectively aggregate important features, we provide a brand-new multi-head linear attention mechanism [23]. As shown in Fig 5. The multiple linear attention mechanism uses a linear sum operation to calculate the attention score, which not only reduces the computational complexity but also improves the numerical stability and consistency of the results.

The input vector is initially transformed into an attention vector of n heads via a linear attention layer, as seen in Fig 5, and then the markers in the dashed box are used to do dot-product operations with the corresponding heads, and the result obtained is used as the output of a specific head. The specific calculation of linear attention is shown in Eq (8), where *W* represents the attention weight matrix, and d_k_ signifies the normalization coefficient.


$$L\mathrm{inear}Attention(W,h_{i})=\frac{\exp(\frac{Wh_{i}}{\sqrt{d_{k}}})}{\sum_{j=1}^{m}\exp(\frac{Wh_{j}}{\sqrt{d_{k}}})}$$ (8)

Take the protein input as an example, suppose the original input feature vector of the protein is $\left\{h_{1}^{p},\cdots ,h_{m}^{p}\right\}$, we get the attention weight vectors of n heads corresponding to the input vector through the linear attention layer computation {head_1_,···,head_n_}, and then compute the sum of the n heads corresponding to the input vector separately and define it as $\left\{a_{1}^{p},\cdots ,a_{m}^{p}\right\}$. Finally, we do the dot product operation of $\left\{a_{1}^{p},\cdots ,a_{m}^{p}\right\}$ with the corresponding original input vectors, and sum the result of the operation as the output of the multi-head linear attention layer. The specific calculations are shown in Eqs (9) and (10).


$$head_{j}=LinearAttention(W_{j},h_{i}^{p})$$ (9)

$$o^{p}=\sum_{i=1}^{m}a_{i}^{p}h_{i}^{p}$$ (10)

Three CNN blocks with various scales are used in the AMCNN module to extract protein features. Following that, the acquired features are sent into the multi-head linear attention layer for processing, respectively, assuming that the feature vectors output from the three CNN blocks are $h_{1}^{p}$、 $h_{2}^{p}$ and $h_{3}^{p}$, respectively, and the specific computation process is shown in Eq (11).


$$\begin{array}{l} O_{1}^{p}=MultiheadlinearAttn(h_{1}^{p})\\ O_{2}^{p}=MultiheadlinearAttn(h_{2}^{p})\\ O_{3}^{p}=MultiheadlinearAttn(h_{3}^{p}) \end{array}$$ (11)

Then we get the outputs $O_{1}^{p}$, $O_{2}^{p}$ and $O_{3}^{p}$ computed by the multi-head linear attention mechanism, and finally we connect the outputs of the three multi-head linear attentions before feeding them into the linear layer to get the final output of the AMCNN module $\hat{h}$. As shown in Eq (12).


$$\hat{h}=Linear(Concat[O_{1}^{p},O_{2}^{p},O_{3}^{p}])$$ (12)

## Result and discussion

### Datasets

We conducted experiments on the Davis [24], Kiba [25], Metz [26] and DTC [27] datasets. The dataset was partitioned into six segments randomly, with five designated for training purposes and one for testing, to assure the experiment’s objectivity and accuracy. The dissociation constant (K_d_) serves as a metric to quantify the affinity within the Davis dataset. And, to visually represent the distribution of affinity more effectively, we convert k_d_ to pk_d_, as shown in Eq (13).


$$pK_{d}=-\log_{10}(\frac{K_{d}}{10^{9}})$$ (13)

The converted pk_d_ values were concentrated between 5.0 and 10.8, with larger pk_d_ values representing greater affinity. Table 1 displays detailed information about each dataset.

**Table 1:** Basic information of the four datasets.

Dataset	Compound	Protein	Interaction
Davis	68	442	30056
Kiba	2111	229	118254
Metz	1471	170	35307
DTC	5983	118	67894

**Fig 6:**
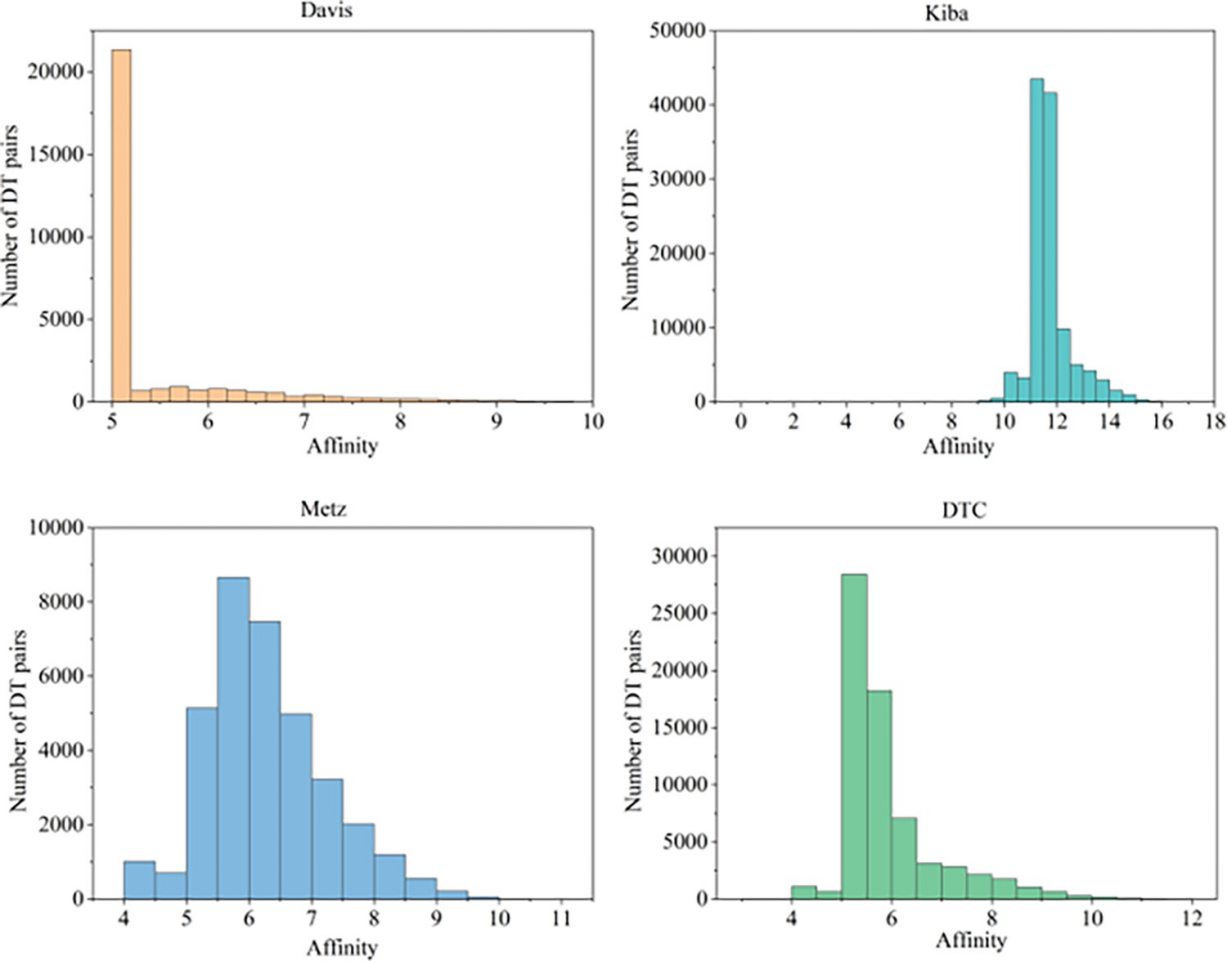
Histograms of affinity distributions for the four datasets.

Fig 6 is a histogram visualizing the affinity distribution of the four datasets.

### Evaluation metrics

As a regression job, DTA prediction uses MSE as the loss function while introducing the consistency index (CI), $r_{m}^{2}$ index, and Pearson to evaluate our model. A lower MSE indicates a tighter match between the expected and actual values, hence a smaller metric is preferable. The MSE is used to quantify the error between the predicted and real values. Eq (14) provides the calculation of the MSE.

$$MSE=\frac{1}{N}\sum_{i=1}^{N}{\left(y_{i}-p_{i}\right)}^{2}$$ (14)

where y_i_ and p_i_ stand for the *i*th sample’s real and predicted values, respectively.

The disparity between the predicted values of two randomly chosen drug-target pairings is assessed using the consistency index (CI) [28], where a higher CI signifies improved predictive performance of the model. The calculation is shown in Eq (15).

$$CI=\frac{1}{z}\sum_{y_{i}>y_{j}}h(p_{i}-p_{j})$$ (15)

where Z is a normalization constant, p_i_ represents the predicted value corresponding to the larger affinity y*i*, while p_j_ represents the predicted value corresponding to the smaller affinity y_j_. Eq (16) illustrates the definition of h(x), a step function [29].


$$h(x)=\{\begin{array}{l} \;0,\;if\;x<0\\ 0.5,\;if\;x=0\\ \;1,\;if\;x>0 \end{array}$$ (16)

An external measure of the model’s prediction ability is the regression toward the mean ($r_{m}^{2}$ index). Specifically, if a variable is large in one measurement, then $r_{m}^{2}$ indicates how close it is to the mean in the next measurement. Generally, when $r_{m}^{2}$ > 0.5, the model’s predictions are considered valid. The $r_{m}^{2}$ index is calculated as shown in Eq (17).

$$r_{m}^{2}=r^{2}\times (1-\sqrt{r^{2}-r_{0}^{2}})$$ (17)

where the correlation coefficients with intercepts and without intercepts are denoted by the symbols r and r_0_, respectively.

Eq (18) is used to compute the Pearson correlation coefficient, where σ(p) and σ(y) are the standard deviation of the predicted value p and the real value y, respectively, and cov(p, y) refers to the covariance between the predicted value p and the true value y. Higher Pearson correlation coefficients mean higher predictive accuracy of the model.


$$Pearson=\frac{\mathrm{cov}(p,y)}{\sigma (p)\sigma (y)}$$ (18)

### Parameter setting

NVIDIA GeForce GTX 4070 12G was the GPU we utilized in our studies. Table 2 shows the precise parameter settings. We fixed the protein’s sequence length to 1000, the number of drug node features to 78, the batch size to 512, the learning rate of the ADAM optimizer to 0.0005, and the dropout rate to 0.2.

**Table 2:** Experimental hyperparameter settings.

Parameters	Setting
Epoch	2000
Batch size	512
Learning rate	0.0005
Optimizer	Adam
Dropout rate	0.2
Number of blocks for CNN and GCN	(3,3)
Number of power graph blocks	(A, A^2^, A^3^)
Number of heads of the multi-head linear attention mechanism	8

### Comparing performance with the baseline model

We used the Davis, Kiba, Metz, and DTC benchmark datasets to evaluate MAPGraphDTA, in order to assess its performance. We compared MAPGraphDTA with KronRLS, SimBoost, DeepDTA, WideDTA, GANsDTA [30], GEFA [31], MATT-DTI [32], GraphDTA, WGNN-DTA, SubMDTA [33] and GLCN-DTA [34]. These models are the more widely used and more advanced DTA prediction models, in order to make certain that the comparison is fair, for our studies, we employed the identical training and test sets, and the experimental results were uniformly evaluated using MSE, CI, $r_{m}^{2}$ and Pearson correlation coefficient.

Table 3 presents our model MAPGraphDTA’s predictive performance against other baseline models using the Davis and Kiba datasets. Bolding indicates the best results and underlining indicates sub-optimal results. As depicted in the table, our model attains superior performance on both the Davis and Kiba datasets. Compared to GraphDTA, which has previously been widely used as a baseline model for DTA prediction, our model showed a decrease in MSE of 11.4% and 11.5% and an increase in CI of 1.0% and 1.7% for the Davis and Kiba datasets, respectively, as well as an increase in $r_{m}^{2}$ of 11.1% for the Davis dataset. Compared to the second best performing baseline model, WGNN-DTA, our model showed a decrease in MSE of 2.4% and 14.6%, an increase in CI of 0.2% and 2.1%, and an increase in $r_{m}^{2}$ of 4.2% and 4.1% on the Davis and Kiba datasets, respectively. Moreover, our model achieved the highest Pearson correlation coefficient, indicating its strong predictive capabilities.

**Table 3:** Model performance on the datasets of Davis and Kiba.

Dataset	Davis				Kiba			
Method	MSE↓	CI↑	$r_{m}^{2}↑$	Pearson↑	MSE↓	CI↑	$r_{m}^{2}↑$	Pearson↑
KronRLS	0.379	0.871	0.407	-	0.411	0.782	0.342	-
SimBoost	0.282	0.872	0.644	-	0.222	0.836	0.629	-
DeepDTA	0.261	0.878	0.630	-	0.194	0.863	0.673	-
WideDTA	0.262	0.886	-	0.820	0.179	0.875	-	0.856
GANsDTA	0.276	0.881	0.653	-	0.224	0.866	0.775	-
GEFA	0.228	0.893	-	0.847	-	-	-	-
MATT-DTI	0.227	0.891	0.683	-	0.150	0.889	0.756	-
GraphDTA	0.229	0.893	0.649	-	0.139	0.889	-	-
SubMDTA	0.218	0.894	0.719	-	0.129	0.898	0.793	-
GLCN-DTA	0.215	0.903	0.720	-	0.127	0.899	0.792	-
WGNN-DTA(GCN)	0.208	0.900	0.692	0.861	0.144	0.885	0.781	0.888
**MAPGraphDTA**	**0.203**	**0.902**	**0.721**	**0.866**	**0.123**	**0.904**	**0.813**	**0.905**

↑means that the higher the evaluation indicator, the better, and ↓means that the lower the evaluation indicator, the better. The blank portion of the table where no data is shown is due to the fact that the original paper referenced in the comparison experiment did not provide data for that portion, making it impossible to obtain the appropriate information.

Table 4 presents our model MAPGraphDTA’s prediction performance against other baseline models using the Metz and DTC datasets. As shown in the table, our model exhibits good prediction performance on both Metz and DTC datasets. Compared to other baseline models, MAPGraphDTA obtained optimal results on all three evaluation metrics.

**Table 4:** Model performance on Metz and DTC datasets.

Dataset	Metz			DTC		
Method	MSE↓	CI↑	$r_{m}^{2}↑$	MSE↓	CI↑	$r_{m}^{2}↑$
GraphDTA(GCN)	0.317	0.801	0.620	0.317	0.878	0.812
GraphDTA(GAT)	0.393	0.775	0.549	0.195	0.859	0.788
GraphDTA(GIN)	0.317	0.800	0.645	0.176	0.876	0.798
GraphDTA(GAT-GCN)	0.333	0.795	0.602	0.200	0.857	0.790
**MAPGraphDTA**	**0.283**	**0.813**	**0.671**	**0.143**	**0.903**	**0.836**

Tables 3 and 4 illustrate the superior performance of our model across all four benchmark datasets, highlighting its remarkable prediction accuracy and stability. Graph neural network-based methods are superior to traditional CNN-based methods because representing compounds as graphs gives access to structural information about the molecules, which leads to richer feature representations. Comparing our model to GraphDTA and WGNN-DTA, which are based on graph neural networks, our performance still shows significant improvement. In GraphDTA, the model extracts drug features using one GCN block and target features using one 1D CNN. In WGNN-DTA the model represents both the drug and the protein as graphs and then extracts the feature information using one GCN block each. In our model, we use three GCN blocks of different scales paired with a gated skip connection mechanism to extract drug features in order to obtain richer and deeper information, and we also introduce the idea of power graphs. For proteins, we use a multi-scale convolutional layer combined with a multi-head linear attention mechanism, which gives us richer and more accurate feature information, and therefore our model attains the highest level of performance.

**Fig 7:**
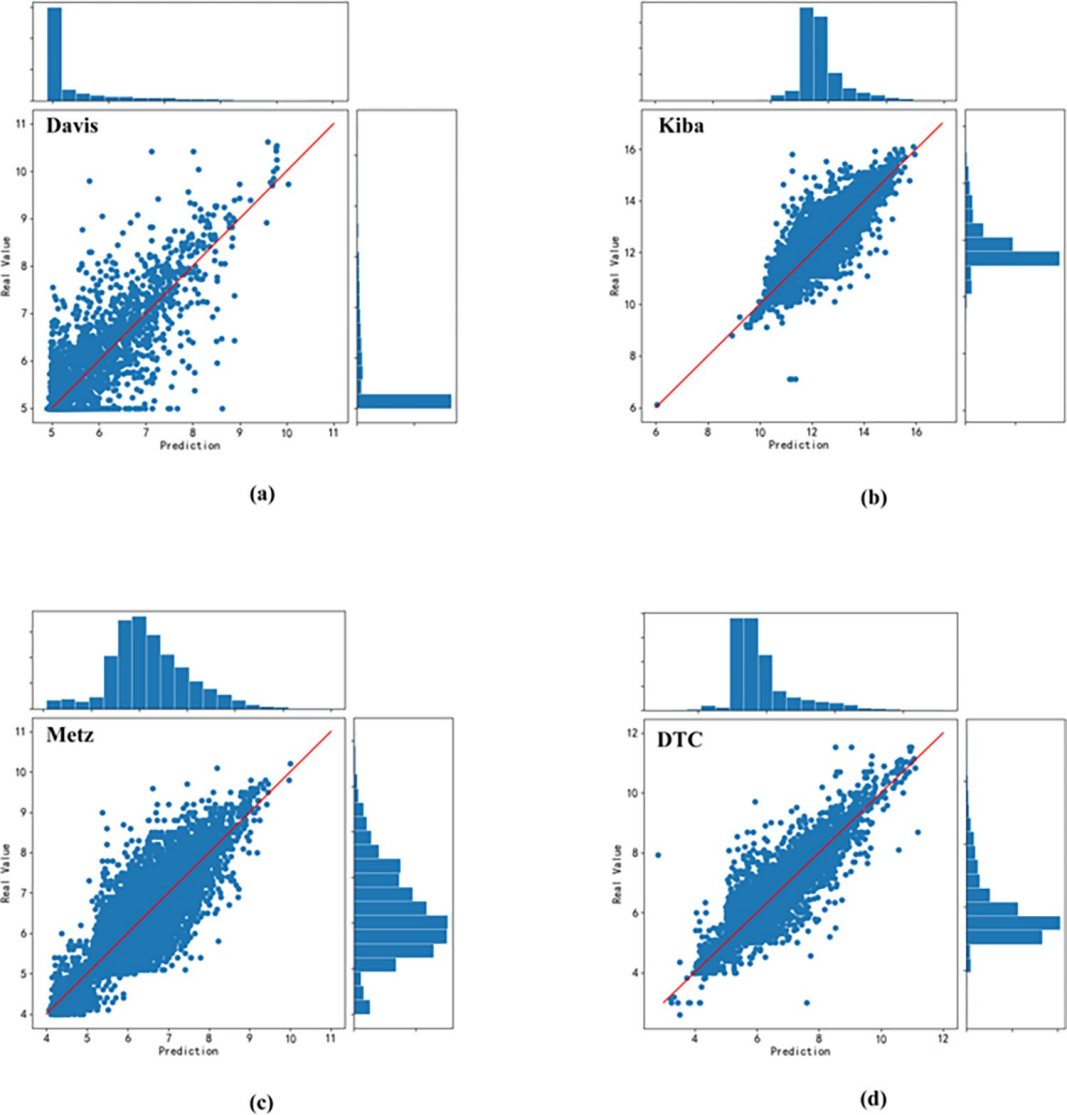
Scatterplot of predicted and true values on Davis, Kiba, Metz and DTC datasets.

Fig 7 illustrates the scatter plots of our model’s real and predicted values across the four benchmark datasets: Davis, Kiba, Metz, and DTC. The anticipated values are shown by the x-axis, while the true values are represented by the y-axis. Additionally, histograms at the edges provide an overview of the distribution of both predicted and true values. When a data point coincides with the line y = x on the scatter plot, it indicates that the anticipated value is exactly identical to the real value. As shown in the figure, the sample points are all closely distributed in the neighborhood of the straight line y = x, indicating that the predicted values roughly align with the real values, and according to the edge histograms, it is evident that the overall distribution of the predicted values closely mirrors that of the true values, which further verifies that our model demonstrates excellent predictive performance.

### Validation of the effectiveness of multi-head linear attention mechanisms

In order to extract protein target feature information more accurately, we introduce a MHLA mechanism in the protein feature extraction module AMCNN, it enhances the prediction performance of the model by selecting aggregating significant feature information. To verify the efficiency of this mechanism, we compare the multi-head linear attention mechanism with several traditional pooling methods, and Table 5 shows the results of our model on the Davis dataset for the multi-head linear attention mechanism compared with several other pooling methods.

**Table 5:** Cold start experiments on the Davis dataset.

Scenario	Method	MSE↓	CI↑	$r_{m}^{2}↑$
Drug cold-start	GraphDTA	0.920	0.678	0.160
	GLFA	0.861	0.670	-
	GEFA	0.847	0.709	0.182
	**MAPGraphDTA**	**0.582**	**0.744**	**0.262**
Target cold-start	GraphDTA	0.510	0.729	0.154
	GLFA	0.453	0.780	-
	GEFA	0.433	0.759	0.289
	**MAPGraphDTA**	**0.408**	**0.828**	**0.493**
Drug-target cold-start	GraphDTA	0.968	0.579	0.026
	GLFA	1.144	0.636	-
	GEFA	0.944	0.610	0.032
	**MAPGraphDTA**	**0.708**	**0.641**	**0.063**

**Fig 8:**
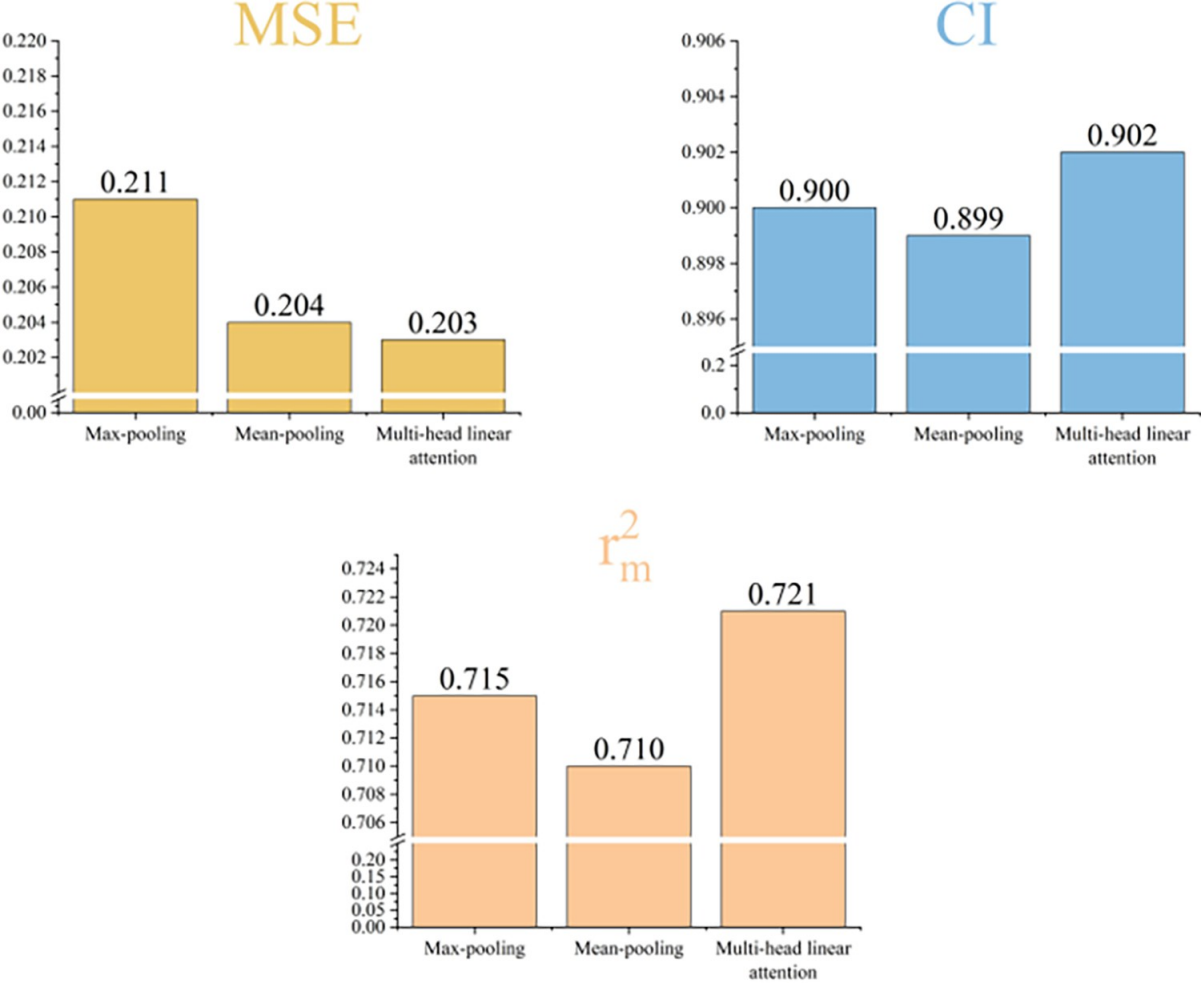
Validation of the effectiveness of the multi-pronged linear attention mechanism.

To assess the influence of various methods on performance, we have all the same model parameters in the experimental stage except for the different pooling methods, which ensures the fairness of the experiment. As shown in Fig 8, we present the performance of the max-pooling, average-pooling, and multi-head linear attention mechanisms. The multi-head linear attention mechanism outperforms the other two pooling techniques, as shown by its higher performance across all three assessment measures.

### Cold start performance

Cold starts are predominantly employed to assess a model’s performance when presented with unknown inputs, essentially evaluating whether the model’s predictions for drugs or proteins not included in the training set remain accurate. For drug discovery purposes, our model needs to have the ability to predict undiscovered drugs, undiscovered proteins, and undiscovered drug-protein pairs; therefore, we used three dataset splits in the cold-start experimental stage: drug cold-start, protein cold-start, and drug-protein cold-start. We take the drug cold-start dataset splitting as an example, we randomly allocate certain drugs to the test set, while assigning the remaining drugs to the training set. Notably, drugs included in the test set are entirely excluded from the training set, thereby achieving drug cold-start splitting. Similarly, in protein cold-start splitting, the training set contains no proteins at all that are chosen for the test set. In drug-protein cold-start splitting, both drugs and proteins included in the test set are entirely excluded from the training set.

We compared MAPGraphDTA with GraphDTA, GLFA and GEFA on the Davis dataset. Table 5 presents a comparison of the performance of drug cold-start, protein cold-start, and drug-protein cold-start. The table illustrates how, in all assessment measures under the three cold-start scenarios of the Davis dataset, our model performs much better than other comparable baseline models, indicating the strong stability of our model in the face of a novel environment.

## Ablation study

To examine the impact of the individual modules within the model on prediction performance, we performed a number of ablation studies using the Davis dataset with power graphs, multi-headed linear attention mechanisms, and gated skip connection mechanisms. We split our experiments into three cases, the first of which removes the multi-headed linear attention mechanism and replaces it with traditional max-pooling. The second scenario is to remove the gated skip connection mechanism. The third case is to split the power graphs and use primary, quadratic and cubic power graphs respectively for comparison experiments, which is equivalent to replacing the power graph module with a normal 1D GCN module when only primary power graphs are used. In all three cases above, the modules and parameters remain unchanged except for the deleted module. Following the experimental validation of the ablation scenarios mentioned above, we can intuitively grasp the extent of influence of each functional module on the predictive performance of the model. Table 6 presents the outcomes of the experiment.

**Table 6:** Performance comparison of ablation experiments on the Davis dataset.

Model	MSE↓	CI↑	$r_{m}^{2}↑$
Without Multi-head linear attention	0.211	0.900	0.715
Without Skip-connection	0.209	0.902	0.713
Without (A^2^,A^3^)	0.208	0.894	0.711
Without (A,A^3^)	0.205	0.898	0**.722**
Without (A,A^2^)	0.208	0.900	0.715
**MAPGraphDTA**	**0.203**	**0.902**	0.721

Table 6 illustrates that the predictive ability of the model notably decreases upon the removal of certain functional modules. The experiments show that all these mechanisms are very important for our model, especially when the multi-head linear attention mechanism is removed, the model’s prediction accuracy experiences a significant decline, underscoring the crucial role played by the mechanism in our model, considering that because the multi-head linear attention mechanism selectively aggregates important feature information according to different weights in the process of feature aggregation, so it is more important for the performance of the model to be of the model is more helpful. When the gated skip connection mechanism is removed, the performance of both MSE and $r_{m}^{2}$ decreases to different degrees, indicating that this mechanism selectively aggregates information from remote nodes and its own nodes, which is also beneficial to the performance of this model. Based on the experimental results of the split power graph module, we can find that for molecular structure graphs, considering multi-hop connectivity relationships can obtain more global and rich information, and therefore can further enhancing the prediction performance of the model.

## Case study

We carried out a case study to evaluate our model’s generalizability. As a validation set for the model, we compiled a collection of drug data from the DrugBank [35] database, where all the drugs are FDA-approved. To enhance the objectivity of the prediction results, we compared this set of drug data with the Kiba dataset, removing drugs that had appeared in the Kiba dataset. We chose a protein epidermal growth factor receptor as the target for this group of drugs and then performed experiments on models already trained on the Kiba dataset. We sorted the affinity magnitudes predicted by the model in descending order and then selected the top 10 potential drugs with the highest predicted affinity for presentation. Table 7 demonstrates the specific information of the top 10 drugs with the highest predicted results.

**Table 7:** Ranking of predicted affinity magnitude of drug candidates.

Rank	Drug_name	Predict_affinity
1	**Erlotinib**	14.34046
2	Icotinib	13.77009
3	**Lapatinib**	13.69808
4	**Gefitinib**	13.41968
5	Olmutinib	13.10224
6	Ibrutinib	13.02137
7	Hesperetin	12.95064
8	**Vandetanib**	12.93876
9	**Afatinib**	12.80249
10	Clavulanic acid	12.79167

**Fig 9:**
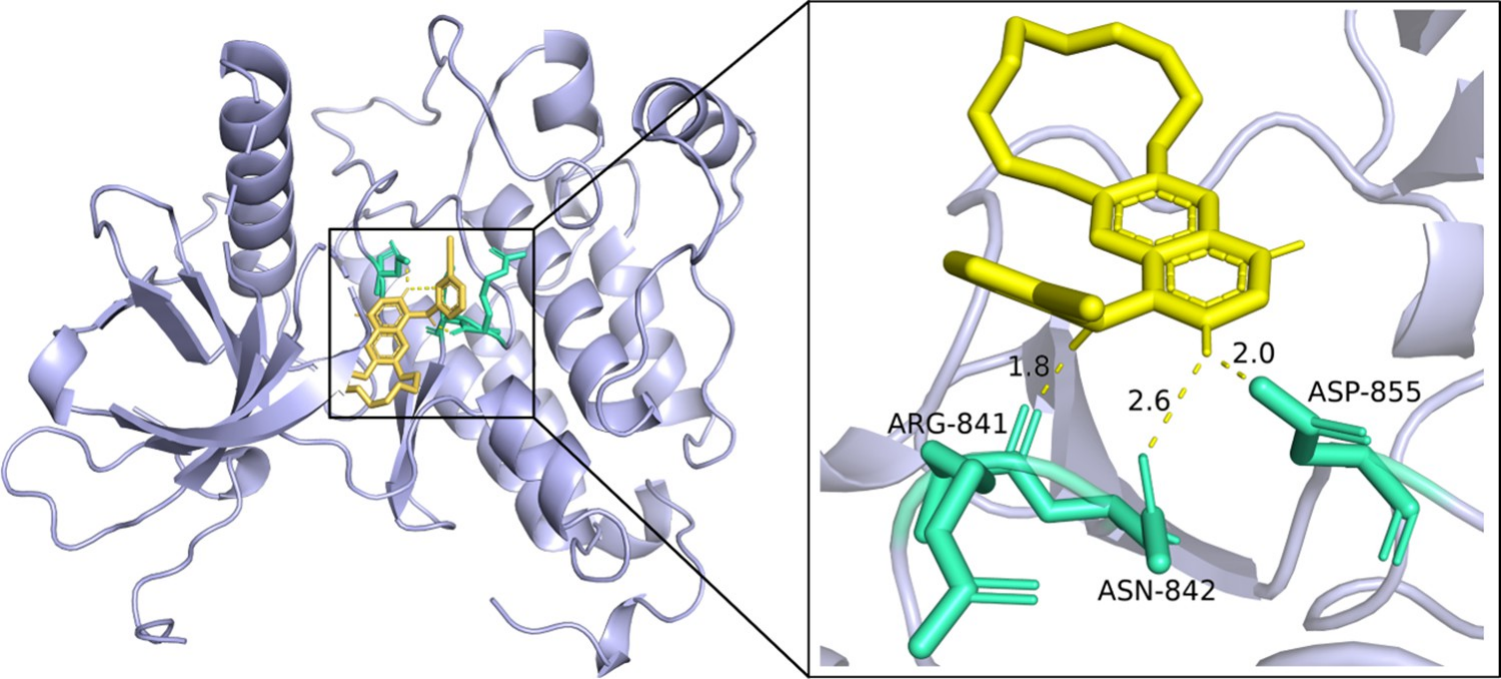
Molecular docking and hydrogen bonding staining results of 3UG2 with Icotinib.

Drug names bolded in Table 7 indicate validated inhibitors of the protein epidermal growth factor receptor, and 5 of the top 10 are known inhibitors, indicating good predictive performance of our model. We downloaded the protein structure 3UG2 (PDB ID) of the protein epidermal growth factor receptor in the PDB database, and then we molecularly docked it to the 2nd ranked drug Icotinib (DB11737) using Autodock [36]. Using Pymol, we analyzed and visualized the structure after selecting the molecular docking binding site with the lowest affinity energy, the visualization is shown in Fig 9.

## Conclusion

We propose MAPGraphDTA, a novel DTA prediction model, in this study, in which we use three GCN blocks with gated skip connection mechanism for extracting drug power graph features and multi-scale CNN with multi-head linear attention mechanism for extracting protein features. This allows us to obtain deeper and more comprehensive feature information. Four benchmark datasets were used in our series of experiments: Davis, Kiba, Metz, and DTC. The outcomes reveal our model’s superior prediction ability, outperforming other state-of-the-art models by a wide margin. We also performed a cold-start performance test on the Davis dataset, which demonstrated the good stability of our model for drug discovery. To validate the generalization ability of our model, we also performed DTA prediction on a dataset of FDA-approved drugs and selected the highest scoring unknown inhibitors for molecular docking and visualization. Overall, our model MAPGraphDTA shows strong performance in DTA prediction as well as unknown drug discovery.

## Supporting information

S1 Dataset (CSV)

## References

[1] Xue H, Li J, Xie H, et al. Review of drug repositioning approaches and resources. International journal of biologic- al sciences, 14(10): 1232, 2018 doi: 10.7150/ijbs.24612 30123072 PMC6097480

[2] Paul S M, Mytelka D S, Dunwiddie C T, et al. How to improve R&D productivity: the pharmaceutical industry’s grand challenge. Nature reviews Drug discovery, 9(3): 203–214, 2010. 20168317 10.1038/nrd3078

[3] Yang Z, Zhong W, Zhao L, et al. MGraphDTA: deep multiscale graph neural network for explainable drug-target binding affinity prediction. Chemical science, 13(3): 816–833, 2022. doi: 10.1039/d1sc05180f 35173947 PMC8768884

[4] Pahikkala T, Airola A, Pietilä S, et al. Toward more realistic drug-target interaction predictions. Briefings in bioinformatics, 16(2): 325–337, 2015. doi: 10.1093/bib/bbu010 24723570 PMC4364066

[5] He T, Heidemeyer M, Ban F, et al. SimBoost: a read-across approach for predicting drug-target binding affinities using gradient boosting machines. Journal of cheminformatics, 9(1): 1–14,2017. doi: 10.1186/s13321-017-0209-z 29086119 PMC5395521

[6] Chu Z, Huang F, Fu H, et al. Hierarchical graph representation learning for the prediction of drug-target binding affinity. Information Sciences, 613: 507–523,2022.

[7] Zhao B W, Su X R, Yang Y, et al. Regulation-aware graph learning for drug repositioning over heterogeneous biological network[J]. Information Sciences, 2025, 686: 121360.

[8] Zhao B W, Su X R, Hu P W, et al. iGRLDTI: an improved graph representation learning method for predicting drug–target interactions over heterogeneous biological information network[J]. Bioinformatics, 2023, 39(8): btad451. 37505483 10.1093/bioinformatics/btad451 PMC10397422

[9] Zhao B W, Wang L, Hu P W, et al. Fusing higher and lower-order biological information for drug repositioning via graph representation learning[J]. IEEE Transactions on Emerging Topics in Computing, 2023, 12(1): 163–176.

[10] Zhu Z, Zheng X, Qi G, et al. Drug–target binding affinity prediction model based on multi-scale diffusion and interactive learning[J]. Expert Systems with Applications, 2024, 255: 124647.

[11] Zhu Z, Yao Z, Qi G, et al. Associative learning mechanism for drug‐target interaction prediction[J]. CAAI Transactions on Intelligence Technology, 2023, 8(4): 1558–1577.

[12] Zhu Z, Yao Z, Zheng X, et al. Drug–target affinity prediction method based on multi-scale information interaction and graph optimization[J]. Computers in Biology and Medicine, 2023, 167: 107621. 37907030 10.1016/j.compbiomed.2023.107621

[13] Öztürk H, Özgür A, Ozkirimli E. DeepDTA: deep drug-target binding affinity prediction. Bioinformatics, 34(17): 821–829,2018. doi: 10.1093/bioinformatics/bty593 30423097 PMC6129291

[14] Öztürk H, Ozkirimli E, Özgür A. WideDTA: prediction of drug-target binding affinity. ArXiv. 2019. 10.1093/bioinformatics/bty593 PMC6129291 30423097

[15] Nguyen T, Le H, Quinn T P, et al. GraphDTA: Predicting drug-target binding affinity with graph neural networks. Bioinformatics, 37(8): 1140–1147, 2021. doi: 10.1093/bioinformatics/btaa921 33119053

[16] Jiang M, Li Z, Zhang S, et al. Drug-target affinity prediction using graph neural network and contact maps. RSC advances, 10(35): 20701–20712, 2020. doi: 10.1039/d0ra02297g 35517730 PMC9054320

[17] Jiang M, Wang S, Zhang S, et al. Sequence-based drug-target affinity prediction using weighted graph neural networks. BMC genomics, 23(1): 1–17, 2022. 35715739 10.1186/s12864-022-08648-9 PMC9205061

[18] Weininger D. SMILES, a chemical language and information system. 1. Introduction to methodology and encoding rules. Journal of chemical information and computer sciences, 28(1): 31–36, 1988

[19] Landrum G. Rdkit documentation. Release, 1(1–79): 4, 2013.

[20] Mukherjee S, Ghosh M, Basuchowdhuri P. DeepGLSTM: deep graph convolutional network and LSTM based approach for predicting drug-target binding affinity. Proceedings of the 2022 SIAM international conference on data mining (SDM). Society for Industrial and Applied Mathematics, 729–737, 2022.

[21] Kipf, T. N, Welling, M. Semi-supervised classification with graph convolutional networks. arXiv preprint arXiv:1609.02907, 2016.

[22] Ryu S, Lim J, Hong S H, et al. Deeply learning molecular structure-property relationships using attention-and gate-augmented graph convolutional network. arXiv. 2018.

[23] Yuan W, Chen G, Chen C Y C. FusionDTA: attention-based feature polymerizer and knowledge distillation for drug-target binding affinity prediction[J]. Briefings in Bioinformatics, 23(1): bbab506, 2022. 34929738 10.1093/bib/bbab506

[24] Davis M I, Hunt J P, Herrgard S, et al. Comprehensive analysis of kinase inhibitor selectivity. Nature biotechnology, 29(11): 1046–1051, 2011. doi: 10.1038/nbt.1990 22037378

[25] Tang J, Szwajda A, Shakyawar S, et al. Making sense of large-scale kinase inhibitor bioactivity data sets: a comparative and integrative analysis. Journal of Chemical Information and Modeling, 54(3): 735–743, 2014. doi: 10.1021/ci400709d 24521231

[26] Metz J T, Johnson E F, Soni N B, et al. Navigating the kinome. Nature chemical biology, 7(4): 200–202, 2011. 21336281 10.1038/nchembio.530

[27] Tang J, Ravikumar B, Alam Z, et al. Drug target commons: a community effort to build a consensus knowledge base for drug-target interactions. Cell chemical biology, 25(2): 224–229, 2018. doi: 10.1016/j.chembiol.2017.11.009 29276046 PMC5814751

[28] Gönen M, Heller G. Concordance probability and discriminatory power in proportional hazards regression. Biometrika, 92(4): 965–970, 2005.

[29] Pahikkala T, Airola A, Pietilä S, et al. Toward more realistic drug-target interaction predictions. Briefings in bioinformatics, 16(2): 325–337, 2015. doi: 10.1093/bib/bbu010 24723570 PMC4364066

[30] Zhao L, Wang J, Pang L, et al. GANsDTA: Predicting drug-target binding affinity using GANs. Frontiers in genetics, 10: 1243, 2020. 31993067 10.3389/fgene.2019.01243 PMC6962343

[31] Nguyen T M, Nguyen T, Le T M, et al. Gefa: early fusion approach in drug-target affinity prediction. IEEE/ACM transactions on computational biology and bioinformatics, 19(2): 718–728, 2021. 10.1109/TCBB.2021.3094217 34197324

[32] Zeng Y, Chen X, Luo Y, et al. Deep drug-target binding affinity prediction with multiple attention blocks. Briefings in bioinformatics, 22(5): 1–10, 2021. doi: 10.1093/bib/bbab117 33866349 PMC8083346

[33] Pan S, Xia L, Xu L, et al. SubMDTA: drug target affinity prediction based on substructure extraction and multi-scale features[J]. BMC bioinformatics, 2023, 24(1): 334. 10.1186/s12859-023-05460-4 PMC10485962 37679724

[34] Qi H, Yu T, Yu W, et al. Drug–target affinity prediction with extended graph learning-convolutional networks[J]. BMC bioinformatics, 2024, 25(1): 75. 38365583 10.1186/s12859-024-05698-6 PMC10874073

[35] Wishart D S, Feunang Y D, Guo A C, et al. DrugBank 5.0: a major update to the DrugBank database for 2018. Nucleic acids research, 46(D1): D1074–D1082, 2018. doi: 10.1093/nar/gkx1037 29126136 PMC5753335

[36] Trott O, Olson A J. AutoDock Vina: improving the speed and accuracy of docking with a new scoring function, efficient optimization, and multithreading. Journal of computational chemistry, 31(2): 455–461, 2010. doi: 10.1002/jcc.21334 19499576 PMC3041641

